# Declining trends in sweetness of the diet in the United Kingdom: 2008/9–2018/19

**DOI:** 10.3389/fnut.2025.1521501

**Published:** 2025-03-13

**Authors:** Inga Kutepova, Alison Kamil, Alissa R. Wilson, Colin D. Rehm

**Affiliations:** ^1^Life Sciences, PepsiCo R&D, Reading, United Kingdom; ^2^Life Sciences, PepsiCo R&D, Chicago, IL, United States; ^3^Life Sciences, PepsiCo R&D, Purchase, NY, United States

**Keywords:** sweetness, sweeteners, cross-sectional studies, trends, United Kingdom, the National Diet and Nutrition Survey

## Abstract

Sugar reduction is a major public health priority. Due to the assumed correlation between dietary sweetness and sugars intake, some organizations suggest minimizing dietary sweetness regardless of source. Data describing the trends/patterns in the sweetness of the diet may inform dietary recommendations. This cross-sectional study utilized dietary data from 2008/09 to 2018/19, including 15,655 individuals ≥1.5 year from the United Kingdom's National Diet and Nutrition Survey Rolling Program. Products sweetened with low-calorie sweeteners (LCS) were matched to their sugar-sweetened pair (e.g., regular cola vs. diet cola), which was used to estimate the sugar equivalents from LCS-sweetened products and estimate dietary level sweetness, defined as grams of approximate sugar equivalent (ASE) per day. Foods and beverages that underwent reformulation during the study period through the use of LCS were also identified. From 2008/9 through 2018/19, the ASE of the overall UK diet declined by about 10%. LCS products contributed 13% of ASE. There was evidence of a non-linear trend, with ASE levels relatively stable until 2014/15 and then declining. Overall, the decline in ASE was larger for beverages than foods (ASE values declined 20.7% for beverages vs. 4.4% for foods), although both decreased significantly (*p*-value < 0.01). Dietary sweetness has changed in the UK, due to a combination of consumer behavior, reformulations, policies, public health awareness programs, and media campaigns, emphasizing its multifactorial nature.

## 1 Introduction

Excessive intake of sugars has been linked to an increased prevalence of overweight and obesity attributed to its promotion of positive energy balance. The World Health Organization (WHO) has published dietary recommendations that advise reducing free sugars consumption to < 10 percent of daily calorie intake (1). In the United Kingdom, the Scientific Advisory Committee on Nutrition (SACN) issued its report on carbohydrates and health in 2015, recommending that no more than 5% of total energy intake should come from free sugars (2). An increasing number of countries are implementing measures such as recommendations reformulation programs, food labeling and taxes on sugar-sweetened drinks (3–8). These efforts typically focus on sugar-sweetened beverages which often contribute to a relative, but not absolute, majority of free/added sugars intakes (9, 10).

During the last decade, the UK government has introduced numerous policies and educational initiatives aimed at reducing sugars consumption. Implemented in 2016, the Sugar Reduction Program sought to cut the amount of sugars added to products contributing the most sugars to children's diets by 20%, by 2020 (11). This program is part of a larger sugar reduction strategy, which includes numerous activities including: (1) the implementation of the Soft Drinks Industry Levy in 2018 (11) public health awareness programs (such as Change4Life initiated in 2009 with different activities throughout this time), and (2) an increasing focus on free sugars and its health implications in the mainstream media (12, 13).

There is emerging interest in limiting sweetness of the diet due to the perceived association between dietary sweetness and sugars intake, theoretically leading to overconsumption of calories. Several organizations have recommended limiting the consumption of all sweet-tasting foods and drinks, regardless of the source of the sweetness [i.e., caloric or low-calorie sweeteners (LCS)] (14–18). An inherent preference or natural liking for sweetness is well recognized (19). The relationship between body weight and consumption of sugars, sweeteners, and sweet foods and drinks has been studied, however there are limited data on the association between exposure to total dietary sweetness and weight. At present, while there may be adequate evidence to suggest limiting total sugars intake, there is little scientific justification for recommendations to limit the overall amount of sweetness in the diet (20). According to systematic reviews, sweet taste from dietary sources with low levels of sugars, sweetened with LCS, may not only replace consumption of free sugars, but may also decrease the desire for sweetness from other sources in the short term (21). Global sales of LCS rose in 2007–2019 (22) perhaps indicating an increased role for LCS in dietary sweetness. Some animal studies suggest LCS exposure affects sweet taste perception (23–25), but hypotheses about LCS altering receptor development, glucose sensing, or decoupling sweetness from energy lack strong evidence and remain debated (26–28). Examining population-level dietary sweetness patterns and trends is an important first step in gaining a deeper understanding of the topic of sweet taste.

In the absence of a standardized approach to measuring total dietary sweetness, it is difficult to examine population-level patterns and trends in dietary sweetness including sweet taste coming from LCS (29). One method previously deployed in the Netherlands involved developing a comprehensive taste database where trained sensory panelists in laboratory settings provided input on many foods and beverages (21, 30). This is a resource-intensive approach that may not be globally applicable as the food supply is incredibly complex, and determining the sweetness of a limited number of foods may not translate well to capturing all dietary sources of sweetness. In our previous research, we used an efficient matched pair approach to quantify sugar equivalents from LCS and sugar-sweetened foods and beverages in order to measure the sweetness level of the US diet, which was reported in grams of approximate sugar equivalents (ASE) per day (31). This population-based study found that dietary sweetness in the United States had declined by about 23% in the absence of centralized federal policies to reduce added sugars consumption. The aim of the current study was to extend this population-based approach to estimate sweetness of the UK diet and to provide the assessment of sweetness across all age and sex groups from age 1.5 years and above, using nationally representative National Diet and Nutrition Survey Rolling Program (NDNS RP) data and examining trends in dietary sweetness and sources of sweetness from 2008/9 through 2018/19.

## 2 Materials and methods

### 2.1 Data sources

Dietary data for this study was obtained from the UK National Diet and Nutrition Survey Rolling Program (NDNS RP) 2008/09 to 2018/19 (referred to in some venues as years 1–11 of the NDNS RP). NDNS RP is a continuous cross-sectional survey, designed to represent the UK population age 1.5 years and over, living in private households (32). Samples were stratified by country, ensuring sufficient representation from England, Scotland, Wales, and Northern Ireland. A paper open-text food diary with estimated portion weights was used to gather data over four consecutive days. Participants received instructions on how to complete food diaries and estimate portion sizes from label information, food photographs or common household measures. To ensure that every day of the week was equally represented, the computer-assisted software randomly allocated 4 consecutive days as the food diary recording period, always including 1 weekend day. Survey interviewers made follow-up checks (in person or via telephone); diary records were verified by interviewers and coded by trained coders. The informed consent was obtained from all subjects. Additional details on the study design are available on the NDNS website (33). All individuals completing food diaries were included in the study, resulting in a final sample size of 15,655 (ranging from 1,094 to 1,948 per cycle).

### 2.2 Estimating sweetness of foods and diets

Estimating the sweetness of foods/beverages and subsequent diets requires creating an operational definition of sweetness and adapting the underlying database to accommodate its calculation. Briefly, consistent with previous work, we opted to use sugar-equivalents as the parameterization of food/beverages dietary sweetness. In a diet consisting only of caloric sweeteners, dietary sweetness would be equivalent to total sugars intake, but individuals also consume foods/beverages sweetened with low-calorie sweeteners. NDNS does not explicitly code LCS-containing foods and beverages. To identify those items that did contain LCS, we analyzed the NDNS data file, which details all foods and drinks (*n* = 4,645) consumed by NDNS participants, for products that were labeled as being “low-sugar,” “diet,” “reduced sugar,” “sugar-free,” “sweetener,” “low calorie,” “NAS” (No Added Sugar), “sweetened with” or “artificial sweetener” etc., in an approach consistent with earlier studies (31, 34, 35). Based on product categories and total and free sugars content, we identified one hundred seventy-seven items that satisfied LCS content requirements (31, 36, 37). For branded products, we verified the existence of LCS in the ingredients when applicable. These items are all listed in Supplementary Table S1.

During the study period, several items reduced their sugars content (e.g., reformulation); for instance, numerous juice drinks and other beverages lowered total and free sugars content by adding LCS, but not exclusively using LCS. However, there are also instances of juice drinks on the market that do not include sweeteners while having significantly decreased fruit juice content; thus, we made assessments based on the sugars amount of certain juice drink flavors. Some categories, such as cereals and chocolate, also reduced their total sugars content over the study period; however, the brands reviewed revealed that this reduction was almost always accomplished without the addition of sweeteners.

After identification of products with LCS, the closest similar food or beverage that did not include LCS was then identified for each of these products. For example, for a sugar free energy drink and a yogurt with artificial sweetener, the matching pair of a regular energy drink and a regular yogurt were identified. These matched pairings were then utilized to estimate the gram-per-gram sweetness of the LCS items. A complete listing of all matched pairs for foods primarily sweetened with LCS are provided in Supplementary Table S1. For certain products where sugars content was decreased due to the addition of sweeteners, the original full-sugar formula was utilized as the matched pair. A complete listing of all foods identified as undergoing reformulation through the addition of sweeteners, and the year this occurred, is available in Supplementary Table S2. Apart from artificial sweeteners, all consumed foods have a gram base unit in the NDNS database, meaning that the amount consumed is described in grams. To minimize errors when estimating intake, artificial sweeteners had a base unit based on their form, such as tablet or teaspoon; hence, 0.5 for granulated artificial sweetener would correspond to 0.5 of a teaspoon and not 0.5 grams. Two authors (IK and CDR) were responsible for identifying products sweetened with LCS and identifying matches. The most consumed products with LCS were beverages (e.g., fruit drinks, soft drinks, lemonades, and energy drinks) and tabletop sweeteners, and the most common foods were yogurt and gum. The data were then modeled in terms of sugar equivalents per day (g/d) and will hereafter be referred to as approximate sugar equivalents (ASE). The ASE is the sum of total sugars and approximate sugar equivalents from products containing LCS and a worked example is provided in Supplementary Table S3.

The study approach assumes that the matched pairs were approximately equal in sweetness, which was substantiated for beverages and yogurts in a small-scale sensory study conducted prior to this analysis (see Supplementary materials Section II), in addition to data from sensory testing conducted in the US for 21 pairs of products (31). In the current study, five pairs of diet vs. regular UK market samples (cola, lemonade, juice drink, yogurt, and energy drink) were evaluated by 11 sensory trained and experienced panelists. The items were analyzed for sweetness and other markers using a 15-point Spectrum Scale. Sweetness quality was evaluated using a 10-point scale ranging from “does not match sucrose at all” to “exactly matches sucrose”. Data were analyzed using ANOVA to identify significant differences between the samples and Fisher's Least Significant Difference multiple comparison test was used to indicate which samples differed from each other for the individual attributes assessed (see Supplementary Table S4).

### 2.3 Statistical methods

Survey-weighted mean total sugars, ASE, ASE from LCS sources and percent of ASE from LCS sources were estimated across all years of NDNS. Data from the three latest cycles were used to provide a snapshot of dietary sweetness by socio-demographic group (e.g., age group, sex, region, ethnic group, income, and body mass index category). Because dietary energy intake is highly correlated (*r* = 0.65) with ASE intakes, energy-adjusted values were also calculated. For trend analyses, survey-weighted linear regression models were used to identify any significant linear trends. Non-linear trends were assessed using permutation tests implemented in the Joinpoint Regression Program (38). Briefly, this approach identifies points of inflection where the estimated direction of the trend changed and is useful for identifying non-linear trends. If a non-linear trend was observed (p < 0.05) the inflection point or joinpoint was identified in tables.

To account for factors driving changes in total sugars consumption, secondary analyses were conducted that disaggregated changes in total sugars consumption due to reformulation vs. changes in consumer behavior. Foods and beverages that were reformulated were identified based on reductions in sugars content. Among the most consumed reformulated foods/beverage were lemonade, fruit drink concentrates, energy drinks and selected other carbonated soft drinks (e.g., Irn Bru, 7-Up). The change in sugars consumption due to reformulation vs. other changes in consumer behavior could then be compared to a counterfactual scenario in which sugars consumption did not change over the study period. It is important to note that changes in consumer behavior could be impacted by a combination of personal preferences, prices, product availability, and policy, but it was impossible to differentiate the impact of these drivers from each other with the available data.

All analyses apart from the sensory trial and the Joinpoint regressions were conducted using Stata 16.1 (College Station, TX). Analyses were weighted to represent the general population and account for survey non-response and also accounted for the complex survey design to ensure proper variance estimation.

## 3 Results

### 3.1 Population characteristics and socio-demographic patterns of sweetness

Population characteristics and mean approximate sugar equivalent (ASE) intakes are shown in Table 1. Overall, the estimated ASE of the diet was 96.7 and 113.8 g/d when normalizing on a per 2,000 kcal basis, respectively. Approximately 13% of ASE came from low-calorie sweeteners (LCS). ASE values increased with age through age 65, then declined, but when adjusting for energy, the youngest children had the sweetest diets (ASE: 129 per 2,000 kcal/d) and older adults had the least sweet diets (110.3 per 2,000 kcal/d). The proportion of ASE coming from LCS was highest among adolescents and younger adults (>14%) but was ≥8% in all ages. In crude analyses, the diets of males had significantly higher ASE values, but when accounting for energy, the diets of females were sweeter. Dietary sweetness varied somewhat by country/region, being the highest in the Central/Midlands of England and lowest in Northern Ireland. While the number of non-white survey respondents limits statistical inference by specific ethnic group, the white population had consistently sweeter diets, higher sugars consumption and obtained a greater proportion of their ASE from LCS sources than the non-white population. In crude analyses higher income individuals consumed slightly sweeter diets than lower income individuals, but when accounting for energy these differences were no longer apparent. Crude and energy-adjusted ASE of the diet was not significantly different by BMI category for adults, though the proportion of ASE from LCS increased with higher BMI values. Similar patterns were observed for children/adolescents in terms of BMI.

**Table 1:** Population characteristics of sample and average approximate sugar equivalents [ASE] (g/d), total sugars (g/d) and proportion of ASE from LCS sources in the United Kingdom, 2017–2019.

	***N***	**Mean (SE)**			
		**ASE, g/d (add E-A) column**	**ASE (g/d) per 2,000 kcal/d**	**Total sugars, g/d**	**% of ASE from LCS sources**
Total	3,558	96.7 (1.1)	113.8 (1.0)	84.0 (0.9)	13.1 (0.5)
**Age group**					
1.5–3	306	68.5 (1.7)	129 (2.2)	62.3 (1.6)	9.1 (0.8)
4–10	725	90.4 (1.3)	125.8 (1.4)	80.6 (1.1)	10.7 (0.5)
11–18	683	96.1 (1.8)	116.1 (1.8)	82.4 (1.6)	14.1 (0.7)
19–64	1,392	101.2 (1.6)	112 (1.4)	86.3 (1.4)	14.7 (0.7)
≥65	452	90 (2)	110.3 (2)	82.4 (1.8)	8.4 (0.7)
P-trend		< 0.001	< 0.001	< 0.001	< 0.001
**Sex**					
Male	1,636	103.5 (1.7)	109.3 (1.3)	90.9 (1.5)	12.1 (0.6)
Female	1,922	90.2 (1.3)	118.2 (1.4)	77.3 (1.1)	14.3 (0.7)
*P*-value		< 0.001	< 0.001	< 0.001	< 0.001
**Region**					
England: North	737	93.7 (2.3)	114.0 (2)	79.9 (1.8)	13.8 (1.1)
England: Central/Midlands	511	102.9 (2.5)	119.3 (2.4)	88.6 (2.2)	14.3 (1.3)
England: South/London	1,244	97.3 (1.8)	112.3 (1.5)	86.0 (1.6)	11.3 (0.7)
Scotland	235	92.1 (3.2)	111.3 (3.9)	77.9 (2.6)	14.2 (2.1)
Wales	273	96.7 (3.1)	116.1 (3.5)	83.4 (2.8)	13.2 (1.5)
Northern Ireland	558	91.0 (3)	107.5 (2)	79.3 (2.7)	11.6 (1.1)
*P*-value		0.02	0.009	0.003	< 0.001
**Ethnic group**					
White	3,118	98.6 (1.1)	116.1 (1.1)	85 (1)	13.8 (0.5)
Non-White	433	84.7 (3.3)	99.4 (2.3)	77.9 (3.1)	7.9 (0.8)
*P*-difference		< 0.001	< 0.001	0.028	< 0.001
**Income**					
Lowest tertile	1,043	92.5 (2.1)	115.9 (1.9)	79.8 (1.8)	13.7 (0.9)
Middle tertile	1,015	99.9 (2.2)	115.8 (2)	85.8 (1.9)	14.1 (0.9)
Highest tertile	1,017	100.3 (1.7)	113.1 (1.6)	87.6 (1.5)	12.7 (0.7)
*P*-trend		0.004	0.25	0.001	0.95
**BMI category (adults)**					
Underweight: < 18.5	37	110.5 (14.4)	107.8 (8)	104.7 (14)	5.2
Healthy weight: 18.5–24.9	708	100.6 (2.4)	111.2 (1.8)	90.3 (2.2)	10.1
Overweight: 25.0–29.9	683	98.2 (2.1)	110.1 (1.9)	84.8 (1.8)	13.6
Obese: ≥30	469	98.1 (2.5)	115.9 (2.8)	80.6 (2)	17.9
*P*-trend		0.34	0.18	< 0.001	< 0.001
**BMI category (children/adolescents)** ^a^					
Normal weight	993	91.2 (1.3)	121.1 (1.2)	81.5 (1.1)	10.5
Overweight	220	90.1 (3.3)	125.1 (3.5)	77.9 (2.7)	13.5
Obese	276	92.8 (2.8)	122.4 (3)	76.1 (2.2)	17.9
*P*-trend		0.67	0.49	0.022	< 0.001

^*a*^BMI categories for children/adolescents based on WHO growth charts.

### 3.2 Overall trends

Across the study period, the ASE of the diet declined by about 10% from 2008/9 through 2018-19, but there was evidence of a non-linear trend, with ASE values generally stable from 2008/9 through 2014/15 and declining thereafter. Similar trends were observed for total sugars, with the same inflection point. Over the same period ASE from LCS sources increased from 8 g/d to 12.6 g/d, and there was no evidence of a non-linear trend (Table 2). Patterns were similar when separating the analysis by children/adolescents vs. adults, though the inflection points did differ. Overall, an inflection point in 2012/13 in ASE values was observed for children/adolescents. Among adults the inflection points for ASE and total sugars were observed in 2014/15 and 2013/14, respectively.

**Table 2:** Trends in total sugars, approximate sugar equivalents (ASE) and ASE from low-calorie sweetener (LCS) sources in g/d from 2008–9 through 2018–19, United Kingdom.

**Year**	**Mean (SE)**		
	**Total sugars, g/d**	**ASE, g/d**	**ASE from LCS sources, g/d**
**Total population (**≥**1.5 years)**			
2008–09	97.3 (1.8)	105.3 (1.8)	8.0 (0.5)
2009–10	96.5 (1.9)	104.5 (2.0)	8.0 (0.6)
2010–11	92.2 (1.6)	100.6 (1.8)	8.4 (0.7)
2011–12	95.4 (1.6)	104.5 (1.8)	9.1 (0.7)
2012–13	96.3 (1.6)	105.9 (1.7)	9.5 (0.8)
2013–14	95.4 (1.8)	104.8 (2.0)	9.4 (0.7)
2014–15	92.0 (1.7)^***^	103.7 (2.0)^***^	11.6 (1.0)
2015–16	91.2 (1.8)	102.6 (2.1)	11.3 (0.7)
2016–17	87.4 (2.0)	98.8 (2.1)	11.4 (0.8)
2017–18	82.9 (1.3)	97.0 (1.6)	14.1 (0.9)
2018–19	81.6 (1.5)	94.2 (1.8)	12.6 (0.9)
*P*-linear trend	< 0.001	< 0.001	< 0.001
*P*-non-linear trend	0.006	0.008	0.85
**Children/adolescents (1.5–18 years)**			
2008–09	99.8 (1.9)	108.1 (1.9)	8.2 (0.6)
2009–10	95.3 (1.7)	105.2 (1.8)	9.9 (0.7)
2010–11	94.8 (1.6)	104.1 (1.8)	9.3 (0.7)
2011–12	98.0 (1.9)	107.0 (2.0)	8.9 (0.6)
2012–13	96.6 (2.1)^***^	105.7 (2.1)^***^	9.1 (0.7)
2013–14	92.6 (1.9)	102.0 (1.9)	9.3 (0.6)
2014–15	88.9 (2.0)	100.0 (2.2)	11.1 (0.8)
2015–16	83.8 (1.6)	94.2 (1.8)	10.3 (0.7)
2016–17	80.7 (1.6)	90.6 (1.8)	9.8 (0.6)
2017–18	77.9 (1.4)	89.2 (1.6)	11.2 (0.7)
2018–19	77.5 (1.5)	89.1 (1.8)	11.6 (0.8)
*P*-linear trend	< 0.001	< 0.001	< 0.001
*P*-non-linear trend	0.008	0.011	0.68
**Adults (**≥**19 years)**			
2008–09	96.5 (2.2)	104.5 (2.2)	8.0 (0.7)
2009–10	96.8 (2.4)	104.3 (2.5)	7.5 (0.7)
2010–11	91.5 (2.1)	99.7 (2.3)	8.1 (0.9)
2011–12	94.8 (2.0)	103.9 (2.1)	9.1 (0.9)
2012–13	96.3 (2.0)	105.9 (2.1)	9.6 (1.0)
2013–14	96.3 (2.3)^***^	105.7 (2.5)	9.4 (0.9)
2014–15	92.9 (2.1)	104.7 (2.5)^***^	11.8 (1.2)
2015–16	93.2 (2.3)	104.9 (2.6)	11.6 (0.9)
2016–17	89.1 (2.5)	101 (2.7)	11.8 (0.9)
2017–18	84.2 (1.6)	99.0 (2.0)	14.8 (1.1)
2018–19	82.7 (1.8)	95.5 (2.3)	12.8 (1.1)
*P*-linear trend	< 0.001	< 0.001	< 0.001
*P*-non-linear trend	0.006	0.008	0.85

Asterisks (^***^) indicate join point (inflection point) in trend-line (*p*-value for non-linearity < 0.05).

Data on trends in total sugars and ASE separated by beverages and foods is shown in Table 3. Overall, foods contributed a majority of both total sugars and ASE to the overall diet, but trends differed across the two categories. Briefly, the decline in total sugars and ASE over time tended to be larger for beverages (ASE values declined 20.7% for beverages) as compared to foods (-4.4%), but it declined significantly for both groups (*p*-value for trend < 0.01). For beverages in the total population, an inflection point was observed in 2015/16 whereafter both ASE and total sugars declined. For children, inflection points for beverages were earlier (2012/13). For neither the total population or for children/adolescents and adults separately was a non-linear trend observed for foods. In terms of ASE from LCS, no evidence of linearity was observed, but it did markedly increase for both categories, but more noticeably for beverages (+62%) than foods (+29%).

**Table 3:** Trends in total sugars, approximate sugar equivalents (ASE) and ASE from low-calorie sweetener (LCS) sources in g/d by beverages and foods separately from 2008–9 through 2018–19, United Kingdom.

**Year**	**Beverages**			**Food**		
	**Mean (SE)**			**Mean (SE)**		
	**Total sugars, g/d**	**ASE, g/d**	**ASE from LCS sources, g/d**	**Total sugars, g/d**	**ASE, g/d**	**ASE from LCS sources, g/d**
**Total population (**≥**1.5 years)**						
2008–09	32.9 (1.5)	39.5 (1.5)	6.6 (0.5)	64.3 (1.1)	65.8 (1.1)	1.4 (0.2)
2009–10	30.9 (1.0)	37.8 (1.2)	6.8 (0.6)	65.5 (1.5)	66.7 (1.5)	1.2 (0.2)
2010–11	30.4 (1.1)	37.4 (1.4)	7.0 (0.7)	61.8 (1.2)	63.2 (1.2)	1.3 (0.2)
2011–12	29.9 (0.9)	37.4 (1.1)	7.5 (0.7)	65.5 (1.2)	67.1 (1.2)	1.6 (0.2)
2012–13	30.1 (1.0)	38.2 (1.3)	8.1 (0.8)	66.2 (1.2)	67.7 (1.2)	1.5 (0.2)
2013–14	30.5 (1.3)	38.6 (1.5)	8.1 (0.7)	64.9 (1.3)	66.3 (1.3)	1.3 (0.2)
2014–15	27.9 (1.2)	37.9 (1.5)	10 (0.9)	64.1 (1.3)	65.8 (1.3)	1.7 (0.2)
2015–16	27.3 (1.3)^***^	37.0 (1.5)^***^	9.7 (0.6)	64.0 (1.2)	65.6 (1.3)	1.6 (0.3)
2016–17	25.4 (1.3)	35.1 (1.5)	9.6 (0.7)	62.0 (1.3)	63.7 (1.3)	1.8 (0.2)
2017–18	22.6 (0.8)	34.4 (1.3)	11.9 (0.8)	60.3 (1.0)	62.5 (1.1)	2.2 (0.3)
2018–19	20.6 (0.8)	31.3 (1.2)	10.7 (0.8)	61.0 (1.2)	62.9 (1.3)	1.8 (0.3)
*P*-linear trend	< 0.001	< 0.001	< 0.001	0.001	0.008	0.001
*P*-non-linear trend	0.001	0.004	0.94	0.07	0.09	0.65
**Children/adolescents (1.5–18 years)**						
2008–09	37.3 (1.3)	45.4 (1.4)	8.1 (0.6)	62.5 (1.3)	62.7 (1.3)	0.2 (0)
2009–10	35.1 (1.1)	44.7 (1.3)	9.6 (0.7)	60.2 (1.1)	60.5 (1.1)	0.3 (0.1)
2010–11	34.6 (1.1)	43.7 (1.3)	9.1 (0.7)	60.2 (1.2)	60.4 (1.2)	0.2 (0)
2011–12	36.7 (1.4)	45.4 (1.4)^***^	8.7 (0.6)	61.3 (1.3)	61.5 (1.3)	0.2 (0.1)
2012–13	34.4 (1.4)^***^	43.4 (1.5)	9.0 (0.7)	62.2 (1.4)	62.4 (1.4)	0.1 (0)
2013–14	31.4 (1.1)	40.5 (1.2)	9.1 (0.6)	61.3 (1.3)	61.5 (1.3)	0.2 (0)
2014–15	28.5 (1.2)	39.3 (1.5)	10.8 (0.8)	60.4 (1.4)	60.6 (1.4)	0.2 (0)
2015–16	26 (1)	36.1 (1.2)	10.1 (0.7)	57.8 (1.1)	58.1 (1.1)	0.3 (0.1)
2016–17	24.8 (1.1)	34.2 (1.3)	9.4 (0.6)	55.9 (1.2)	56.4 (1.2)	0.4 (0.2)
2017–18	21.4 (0.9)	32.5 (1.2)	11.1 (0.7)	56.5 (1.2)	56.7 (1.2)	0.2 (0)
2018–19	21 (0.9)	32.4 (1.2)	11.4 (0.8)	56.5 (1.2)	56.8 (1.2)	0.2 (0.1)
*P*-linear trend	< 0.001	< 0.001	< 0.001	< 0.001	< 0.001	0.35
*P*-non-linear trend	0.002	0.002	0.55	0.09	0.08	0.95
**Adults (**≥**19 years)**						
2008–09	31.7 (1.8)	37.9 (1.9)	6.2 (0.6)	64.9 (1.4)	66.6 (1.4)	1.8 (0.2)
2009–10	29.8 (1.3)	35.9 (1.5)	6.1 (0.7)	67.0 (1.9)	68.4 (1.9)	1.5 (0.2)
2010–11	29.2 (1.4)	35.7 (1.7)	6.5 (0.9)	62.3 (1.5)	64.0 (1.5)	1.7 (0.2)
2011–12	28.2 (1.0)	35.4 (1.3)	7.2 (0.9)	66.5 (1.5)	68.5 (1.5)	1.9 (0.3)
2012–13	29.1 (1.2)	36.9 (1.5)	7.8 (1.0)	67.2 (1.4)	69.0 (1.4)	1.8 (0.3)
2013–14	30.2 (1.6)	38.0 (1.9)	7.7 (0.9)	66.0 (1.6)	67.7 (1.6)	1.7 (0.3)
2014–15	27.8 (1.4)	37.5 (1.9)	9.8 (1.1)	65.1 (1.6)	67.1 (1.6)	2 (0.3)
2015–16	27.6 (1.6)^***^	37.2 (1.9)^***^	9.6 (0.8)	65.6 (1.5)	67.6 (1.6)	2 (0.4)
2016–17	25.6 (1.7)	35.3 (1.9)	9.7 (0.9)	63.5 (1.6)	65.7 (1.6)	2.1 (0.3)
2017–18	22.9 (1.0)	35.0 (1.6)	12.1 (1.1)	61.3 (1.3)	64.1 (1.3)	2.8 (0.3)
2018–19	20.5 (0.9)	31.0 (1.4)	10.6 (1.0)	62.2 (1.5)	64.5 (1.6)	2.3 (0.4)
*P*-linear trend	< 0.001	0.048	< 0.001	0.021	0.098	0.002
*P*-non-linear trend	0.002	0.015	0.93	0.13	0.11	0.65

Asterisks (^***^) indicate join point (inflection point) in trend-line (p-value for non-linearity < 0.05).

### 3.3 Trends by food/beverage category

**Figure 1:**
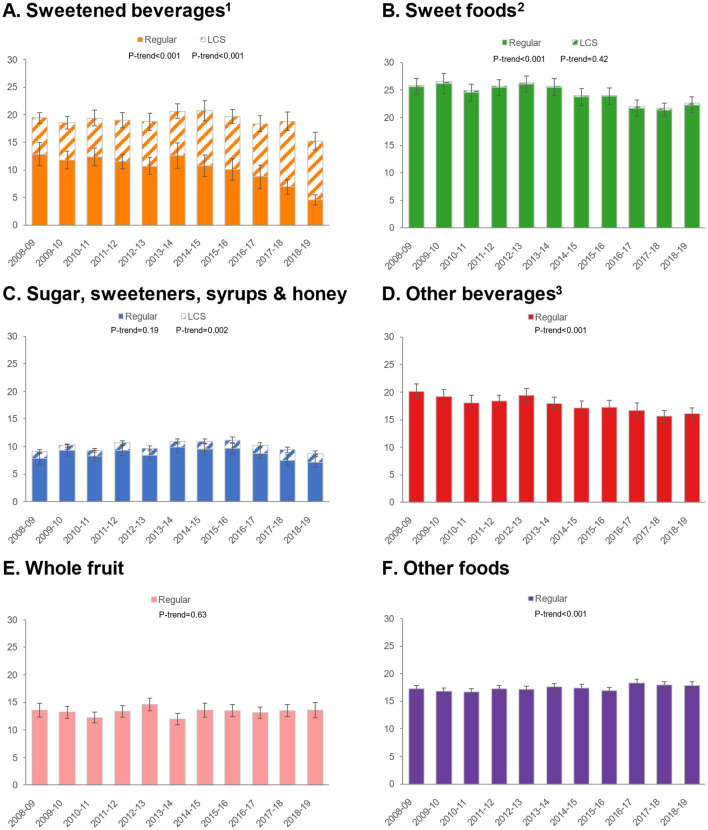
Trends in approximate sugar equivalents (g/d) from regular and low-calorie sweeteners by food/beverage category in the total population. **(A)** Sweetened beverages; **(B)** Sweet foods; **(C)** Sugar, sweeteners, syrups & honey; **(D)** Other beverages; **(E)** Whole fruit; **(F)** Other foods. Error bars are 95% confidence intervals. ^1^Sweetened beverages include carbonated soft drinks, fruit drinks, sports, and energy drinks. ^2^Sweet foods include chocolate confectionary, cakes, puddings, yogurt, biscuits, sugar confectionary, ice cream and sweet ready-to-eat cereals. ^3^Other beverages include milk, 100% fruit juice, tea and alcoholic beverages.

**Figure 2:**
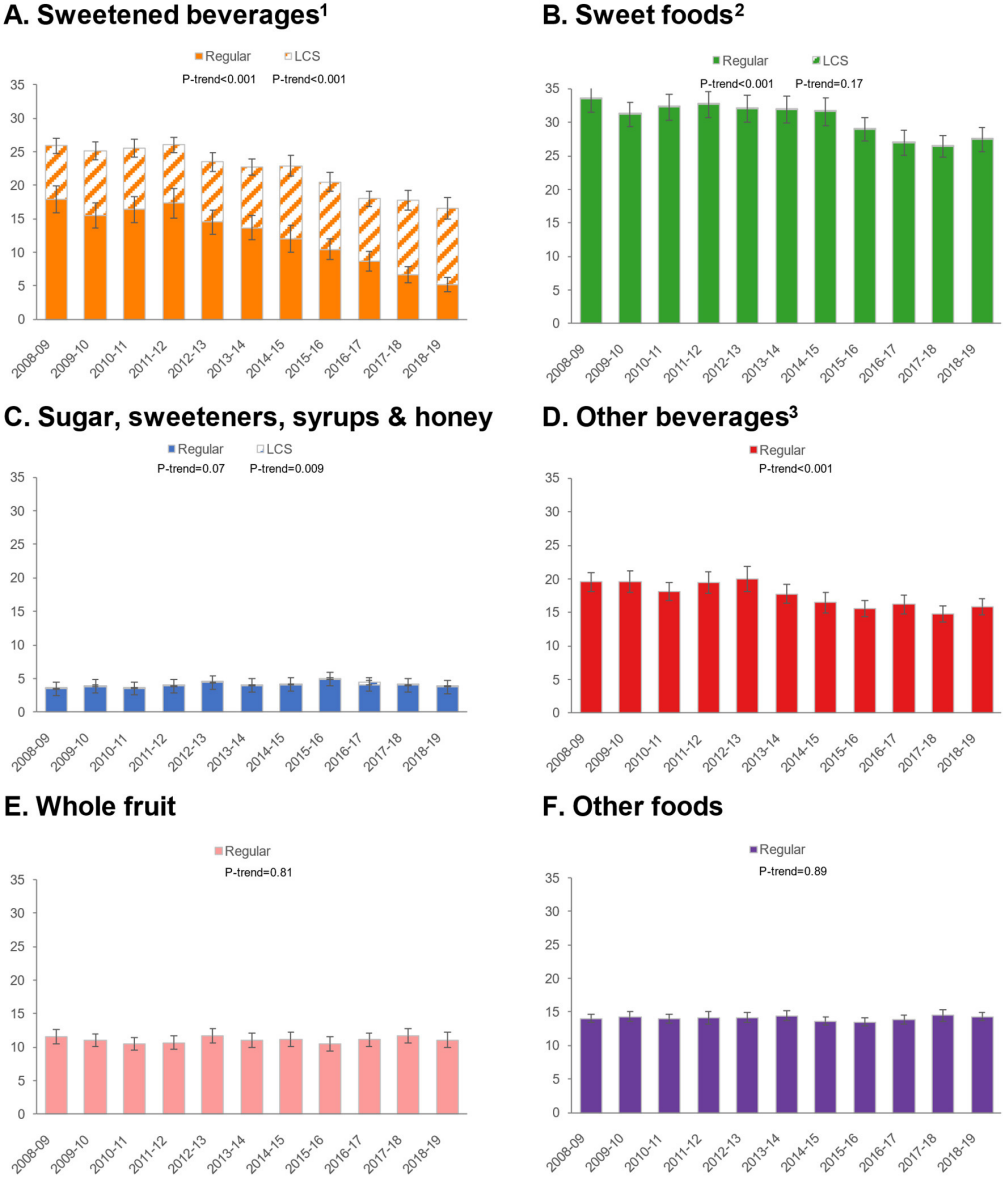
Trends in approximate sugar equivalents (g/d) from regular and low-calorie sweeteners by food/beverage category among children/adolescents. **(A)** Sweetened beverages; **(B)** Sweet foods; **(C)** Sugar, sweeteners, syrups & honey; **(D)** Other beverages; **(E)** Whole fruit; **(F)** Other foods. Error bars are 95% confidence intervals. ^1^Sweetened beverages include carbonated soft drinks, fruit drinks, sports, and energy drinks. ^2^Sweet foods include chocolate confectionary, cakes, puddings, yogurt, biscuits, sugar confectionary, ice cream and sweet ready-to-eat cereals. ^3^Other beverages include milk, 100% fruit juice, tea and alcoholic beverages.

**Figure 3:**
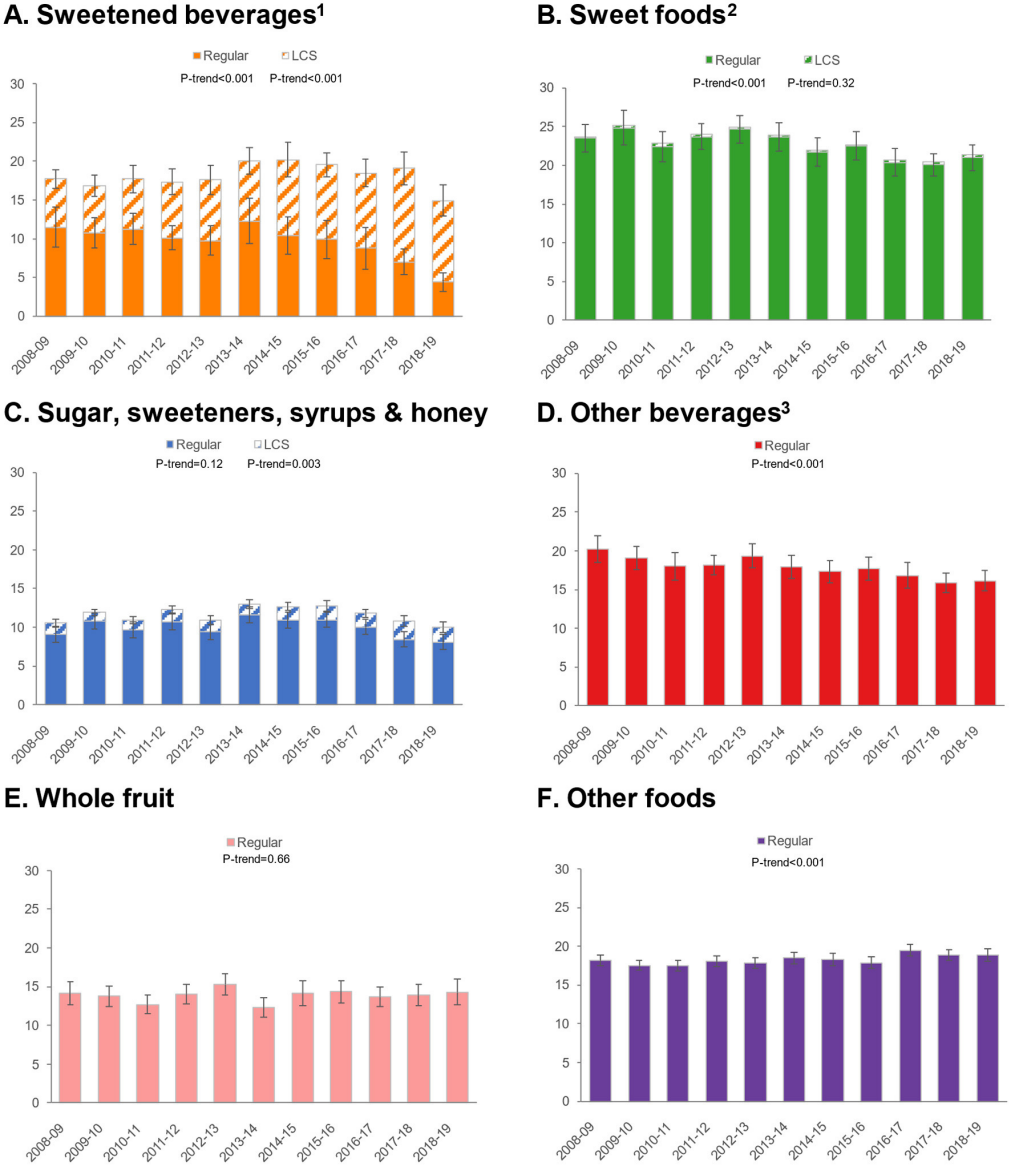
Trends in approximate sugar equivalents (g/d) from regular and low-calorie sweeteners by food/beverage category among adults. Error bars are 95% confidence intervals. **(A)** Sweetened beverages; **(B)** Sweet foods; **(C)** Sugar, sweeteners, syrups & honey; **(D)** Other beverages; **(E)** Whole fruit; **(F)** Other foods. ^1^Sweetened beverages include carbonated soft drinks, fruit drinks, sports, and energy drinks. ^2^Sweet foods include chocolate confectionary, cakes, puddings, yogurt, biscuits, sugar confectionary, ice cream and sweet ready-to-eat cereals. ^3^Other beverages include milk, 100% fruit juice, tea and alcoholic beverages.

Given differential trends in ASE intakes for foods/beverages, we conducted additional analyses to examine trends in ASE for six food and beverage categories: sweetened beverages, sweet foods, sugars/sweeteners/syrups/honey, other beverages, whole fruit, and other foods (Figure 1). For sweetened beverages, total ASE values declined, but an increase was observed in ASE from LCS and a dramatic decrease in sweetness from sugars/caloric sweeteners. ASE from sweet foods declined, but there was practically no contribution of LCS to this category. Similarly, ASE from other beverages declined, and there was no measurable impact of LCS in this category. ASE from whole fruit was unchanged, while ASE from other foods increased slightly. Patterns were similar for children/adolescents (Figure 2) and adults (Figure 3).

### 3.4 Factors driving changes in total sugars

**Figure 4:**
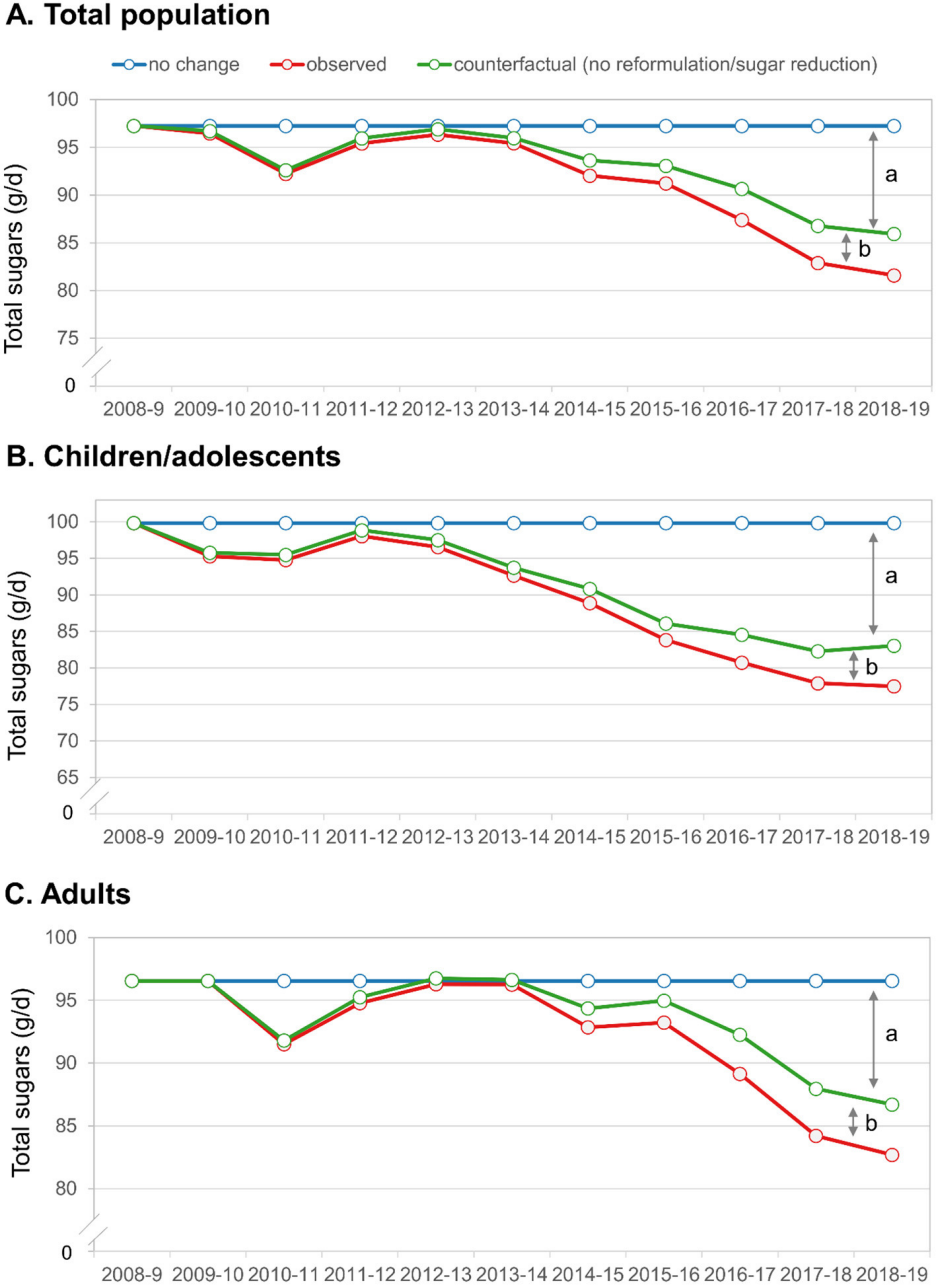
Trends in observed total sugars and change due to reformulation/sugars reduction vs. other factors. **(A)** Total population; **(B)** Children/adolescents; **(C)** Adults. The letters denote the factors explaining the decrease in total sugars. (a) Represents the change due to factors other than reformulation/sugars reduction and (b) represents the change due to reformulation/sugars reduction.

Additional analyses (Figure 4) show trends in overall sugars consumption, plotting the potential change due to reformulation/sugars reduction (e.g., participants reporting consumption of items that were reformulated per NDNS databases) vs. other factors. For all populations, both factors appear to play a role in sugars reduction, but the impact of reformulation/sugars reduction is markedly lower than changes due to other factors, which may include participants selectively choosing lower sugars products, avoiding sugary products, having fewer sugary products available, or consuming less amounts of said products. For children, the impact of reformulation was smaller than for adults.

## 4 Discussion

To our knowledge, this is the first systematic effort to estimate the sweetness of the UK diet. In this study we used a pragmatic approach to estimating the sweetness of the diet and used this methodology to examine socio-demographic patterns and temporal trends in sweetness. Key findings were that the sweetness of the UK diet declined after 2014/15 and was stable prior to that. Trends in total sugars generally mirrored trends in dietary sweetness, but the amount of sweetness from LCS sources increased by about 58%. We did not observe any non-linear trends in sweetness from LCS sources, which appeared to steadily increase, in contrast to total sugars and ASE which only decreased from around 2014/15. The observed trends were qualitatively similar for adults and children/adolescents. Declines in both total sugars and dietary sweetness appear to be mostly driven by changes for beverages, though very small but statistically significant declines in foods were also observed. Specifically, sweetened beverages, which include carbonated soft drinks, fruit drinks, and sports/energy drinks showed the most profound decrease.

As noted, there are few studies examining population-level dietary sweetness patterns and trends, due in part to lack of a consensus methodology to estimating dietary sweetness. Several methods have been employed to evaluate dietary sweetness, but each has limitations, particularly when applied to large-scale studies. One common approach involves the use of trained sensory panels to measure the sweetness intensity of individual food and beverage items in controlled laboratory settings (29, 39–41). These estimates can then be aggregated into taste databases to approximate the sweetness of a diet. While this method has been effectively used, such as in studies of the Dutch and Australian diet, it is resource-intensive and challenging to implement in regions with diverse and dynamic food supplies. Sensory evaluation methods, though well-established, face challenges due to the lack of an absolute reference standard for sweetness, as well as variability in perception based on context, scales, and individual experience. The absence of standardized procedures for measuring the total dietary sweetness makes it difficult to compare findings across studies or regions. Addressing these methodological gaps remains essential for advancing our understanding of sweetness intake on a broader scale. A recent study was conducted utilizing sales data from Euromonitor (2007–2019) to assess the quantity of added sugars and LCS sold in packaged foods and beverages (22). The researchers observed that across countries studied (mostly higher income and upper middle-income countries) the amount of added sugars and LCS combined, also referred to as the total sweetness, experienced a decrease of about 9.7% for beverages, while it increased by 7.6% in packaged foods. Data on the United Kingdom specifically were not provided, but the general trends for beverages, but not foods, observed by Russell et al. are directionally consistent with our results. The approach used in estimating the sales of LCS in grams is limited by several constraints, primarily caused by the lack of available information regarding the specific quantities of LCS indicated in the ingredients list (22). Our prior study employing a similar methodology as in the current research using the National Health and Nutrition Examination Survey (NHANES) revealed decreases in dietary sweetness from 1999 to 2018 (31). Comparing differences and similarities in the socio-demographic patterns of dietary sweetness between the two populations US and UK also reveals some interesting patterns. Specifically, in the UK exposure to LCS appears to be more ubiquitous and less patterned by socio-demographics. Specifically, in the UK while there were small differences observed in ASE from LCS by age group and gender, these differences were much less profound than those observed in the US. Further, we observed no income-gradient in LCS exposure in the UK, whereas there was a strong income gradient in the US. Body mass index results were similar between the UK and US and mirrored an observation from a Dutch study (30). Here we observed that individuals who were obese did not consume sweeter diets than their healthy weight peers but did have the highest consumption of LCS. This pattern likely reflects the preferential use of LCS by heavier individuals who are attempting to manage or lose weight (36).

For trends, results were similar, though declines in both total sugars and ASE in the US were more dramatic than those observed here, perhaps because of differences in the study duration (18 years vs. 11 years here), differences in baseline dietary intakes, or due to different supply and demand factors influencing dietary intakes. In the US we did not observe a non-linear trend, and like the UK changes in both sugars and sweetness from beverages appear to be major driving forces behind these trends, specifically sweetened beverages including carbonated soft drinks and fruit drinks. One notable difference in observed trends was an increase in ASE from LCS in the UK, but a slight decrease in the US, due to lower consumption of LCS sweetened beverages from the late 2000s onward. Notably, in the US, while concern regarding high added sugars consumption has been clearly articulated for some time there have been very limited coherent nationwide policy efforts to influence consumption. Notable policy changes implemented in the US include calorie-labeling at major restaurant chains, limiting sales/availability of competitive foods in schools, and the addition of added sugars to the Nutrition Facts panel, but all changes occurred well after the decline in both total sugars and sweetness were observed.

On the other hand, the UK has taken a much more aggressive approach to population-wide interventions to influence dietary intakes and address obesity. In its 2015 report on Carbohydrates and Health, the Scientific Advisory Committee on Nutrition (SACN) in the UK advised that free sugars should make up no more than 5% of total calorie intake (2, 42). As an example of a government initiative, the Change4Life campaign encouraged parents to reduce their children's sugars intake by adopting easy replacements, such as switching from sugary beverages to sugar-free or no-added-sugars drinks (43). Through voluntary and economic policy measures, the UK government has promoted reformulation to improve public health (44). Public Health England (PHE) oversees the Sugar Reduction Program, which was launched in 2016 as part of the UK Government's Childhood Obesity Strategy and is a continuation of the Public Health Responsibility Deal that was an initiated in March 2011 (11, 45). The Sugar Reduction Program planned to cut the amount of sugars added to those products that contribute the most to children's intake by 20%, targeting items including biscuits, cakes, confectionery, yogurts, breakfast cereals, fruit juices, and milk-based drinks, while “cereals and cereal products”, “non-alcoholic beverages”, and “sugars, preserves, confectionery” were the main sources of free sugars consumption in the UK (11, 46). Between 2015 and 2019, the Sugar Reduction Program's progress was inconsistent, according to the PHE report, varying by industry and product category (47, 48). For instance, the average sugars content of items from the out-of-home (OOH) industry remained constant while that of products bought for consumption at home decreased by 3% on average (47). Breakfast cereals and yogurts showed reductions >10%, whereas confectionery showed essentially no change (47).

From a technical perspective it is theoretically simpler to decrease sugars in beverages than in some foods. In Europe, for a producer to utilize LCS in a particular product, the food or beverage must have a 30% decrease in calories or have no added sugars (Annex II, Regulation 1333/2008 on food additives). The Sugar Reduction Program excludes beverages subject to the Soft Drinks Industry Levy (SDIL). The SDIL imposed a graduated tax on soft drinks containing 5 grams of sugars per 100 mL announced in March 2016 and took effect in April 2018 (11, 49). The SDIL only applies to beverages sweetened with added sugars and excludes fruit juices and milk products. It seeks to encourage producers to voluntarily lower the sugars content of their beverages, and to shift consumers' purchasing patterns toward lower or no added sugars products.

The substitution of added sugars with low-calorie sweeteners is one technique for reducing energy intakes and managing body weight (50). Human randomized controlled trials up to 2 years in length have demonstrated that LCS can aid in weight maintenance when used in place of sugar (51, 52). Maintaining the sweet taste of foods and beverages with fewer calories may be advantageous; for instance, LCS-sweetened products can provide more food and beverage options for those intending to reduce their sugars and calorie intakes and permit the enjoyment of some sweet-tasting products with fewer calories. Moreover, beverages containing LCS were not different from water in effects on appetite, energy intake and food choices (53, 54).

In both of our studies, we observed a decline in total dietary sweetness over the years studied despite generally increasing body weight. Despite this, the question remains as to whether dietary exposure to sweetness in humans influences the subsequent preference, acceptability, and consumption of other sweet products. Unconfirmed beliefs that consumption of sweet items (excluding fruits) trains palates to seek sweetness and contributes to calorie overconsumption result in public health and nutrition policies and recommendations to reduce all sweetness in the diet, regardless of whether it comes from naturally occurring sugars, added sugars, or LCS. A literature review conducted by Public Health England determined that there is insufficient evidence to support the hypothesis that frequent exposure to sweetness might cause habituation to sweet taste (55). Similarly, findings from intervention trials and longitudinal studies suggest that acute exposure to sweetness often reduces subsequent liking, consistent with sensory-specific satiety (56, 57), while sustained exposure has no significant effects or inconsistent outcomes (21, 58–60). Additionally, exposure to higher or lower levels of dietary sweetness does not appear to significantly influence energy intake or body weight, and individual liking for sweetness is not strongly associated with obesity, sugar consumption, or overall diet quality (20, 29, 58, 61–66). A preference for sweet taste alone does not fully explain sugar consumption; factors such as a food's flavor, texture (60) and individual attitudes also contribute significantly to food choices and consumption in real-life contexts. Comprehensive reviews, such as those by Appleton et al. (21) and Venditti et al. (58), further emphasize that the relationship between exposure to sweetness and sweet taste preference remains inconclusive, disputing the assumption that sweetness exposure inherently leads to a stronger preference for sweet flavors. These findings suggest that substituting free sugars with LCS may reduce sugar intake and, in the short term, even suppress the desire for sweetness without reinforcing a long-term ”sweet tooth.“ A recent study explores how a sweet-tasting diet impacts sweetness perception and consumption (67). Reduced sweet food intake heightened sweet taste intensity but did not affect pleasantness, desire, or consumption, highlighting the role of hedonic preferences over perception in sweet food intake. The available evidence predominantly originates from studies conducted in populations in the USA, Western Europe, and Australia. It remains possible that populations with significantly lower or higher habitual consumption of sweetened foods and beverages might exhibit different responses to changes in sweetness exposure.

Studies comparing LCS with sugars indicate similar impacts on sweetness preferences, with no conclusive evidence that LCS consumption disrupts natural sweet taste perception or fosters a greater preference for sweetness. Animal studies (23–25) have suggested that exposure to low-calorie sweeteners (LCS), compared to sugars, might influence the development of sweet taste perception and preferences, with the WHO also highlighting the potential for early LCS consumption to affect later sugar preferences in child-feeding guidance (16). Proposed mechanisms include changes in sweet taste receptor expression, glucose sensing, or ”uncoupling“ sweetness from energy content; however, these hypotheses are supported by limited and often equivocal evidence, with challenges to their replicability and interpretation (26–28). Human trials indicate that LCS generally have minimal effects on physiological responses, such as cephalic phase reactions and gut hormone secretion, and that sweetness is a poor predictor of energy content in diets, even when LCS are excluded (40, 68). Comparisons of LCS and sugars in controlled trials reveal largely similar outcomes on sweet taste preferences, though some inconsistencies arise (59). Overall, the evidence does not strongly support the notion that LCS uniquely disrupt sweetness perception or energy regulation.

In our study, the ASE of the diet decreased by around 10% from 2008/9 to 2018/19, but there was evidence of a non-linear trend, with ASE levels being relatively steady from 2008/9 to 2014/15 and then lowering thereafter. The observed non-linear trends in both total sugars and ASE values merits some discussion, as when these inflection points were observed may offer some clues to what factors proved most impactful. Also, over this period, the UK population consumption of free sugars decreased, although this should not be attributed to the UK sugar levy because it was not implemented until 2018. The current analysis suggests that for whatever reason, UK consumer preferences shifted toward less sweet products, though this trend appears to have emerged prior to the implementation of major sugar reduction policies. While reformulation efforts occurred throughout the study period a bulk of them occurred in 2015/16 and 2016/17, which is just slightly after the infection points we observed (2014/15 for the overall population). For total sugars among children the inflection point was 2013/14 well before most reformulation activities were implemented. Noted above, a wide array of public policy and educational approaches that could impact both total sugars and ASE values have been implemented in the UK in the past 10–15 years. Consumer behavior can be shaped by personal preferences, pricing, product availability, public communications, and policy. With the data on-hand it is not justified to attribute the observed changes and non-linearity to a single action, government report or program. The interplay between reformulation, consumer behavior, and media campaigns is complex and merits further study (69). Reformulation modifies product attributes to align with healthier standards, while media campaigns not only promote healthier choices but also educate on nutrition and foster behavior change. These efforts, working together or independently, influence awareness and preferences. At present, it would be most appropriate to simply acknowledge that the drivers of these changes are multi-factorial.

Given the observed trends, it is clearly of considerable interest to identify the forces driving those trends. This study was not designed to explicitly evaluate this impact of individual interventions, but the changes in dietary sweetness observed here appeared to occur prior to implementation of selected policies (e.g., SDIL, Sugar Reduction Program). Further, our secondary analyses examining trends in dietary sweetness and total sugars consumption due to product reformulation vs. shifting consumer behavior (e.g., stopping consumption of high sugars beverages or shifting to low-calorie versions) shows that most of the impact was due to shifts in consumer behavior, but both factors did play a role in reducing sugars consumption. Over time, this pattern may change as more products are reformulated, especially if commonly consumed items undergo reformulation.

## 5 Limitations and strengths

Like all observational studies of dietary intakes, this study is subject to numerous limitations. Chief among these is the reliance on self-reported dietary data, which is subject to numerous random and systematic errors and measuring exposure to LCS is known to be particularly prone to misclassification as not all subjects may accurately report/recall this information (70). This may include systematic under-reporting intake of some sweet/sugary foods/beverages and errors in the underlying dietary databases that are generalized representations of a complex food system. Given the estimated energy intakes reported in NDNS (1807 kcal/d for adults), under-reporting is occurring. The impact of under-reporting on the assessments of trends would only be a major threat to validity if it was substantially worsening over time; we are not aware of any data implying that this may be occurring. We observe a very small decrease in reported energy intakes after adjusting for age, sex and BMI among adults, but the extent of differences year-over-year was never more than 4%, suggesting a general random pattern and indirect evidence that under-reporting is unlikely to have meaningfully increased. The underlying nutrient databases driving this work were regularly updated and appeared to reasonably represent changes to the food system but may not fully account for all changes. Most importantly, dietary sweetness is a complex construct, and our approach makes numerous simplifying assumptions (29). There are certainly interactions between different dietary components and other organoleptic properties of food that may impact perceived dietary sweetness. We did confirm that, for a very small number of products, none of the tested had sweetness levels that differed from their matched pairs (see Supplementary material). Further, in 21 pairs of products tested in the US, most beverages had similar sweetness levels and there was no consistent pattern of LCS vs. regular items being sweeter (31). That said, developing a taste database for a complex dietary survey is infeasible. Strengths of the study include the use of a nationally representative population and the use of a four-day food record, which can reasonably approximate habitual dietary intakes at the population level. Further, such a study would not be possible when using simplified dietary assessments, such as a food frequency questionnaire, which lacks the level of detail to approximate dietary sweetness.

## 6 Conclusions

This is the first comprehensive attempt we are aware of to assess the sweetness of the UK diet. During 2008/9 to 2018/19, the ASE of the diet declined by around 10%, however there was evidence of a non-linear trend, with ASE levels being relatively stable until 2014/15 and then declining afterwards. Decreases in total sugars and dietary sweetness seem to be mostly attributable to reductions in beverages, while minor but statistically significant decreases in foods were also found. Additionally, over this period, the consumption of total sugars declined, but this should not be directly linked to the UK sugar tax levy, which was not enacted until 2018.

## Data Availability

The original contributions presented in the study are included in the article/Supplementary material, further inquiries can be directed to the corresponding author.
