# Supplementary material for: Declining trends in sweetness of the diet in the United Kingdom: 2008/9–2018/19
Source: Front Nutr. 2025 Mar 13;12:1521501. doi: 10.3389/fnut.2025.1521501 (PMC11948284; doi:10.3389/fnut.2025.1521501)
Supplement: Supplementary file 1 [file Table_1.docx]

Supplementary Material

**Declining trends in sweetness of the diet in the United Kingdom: 2008/9 through 2018/19**

**Part I. Implementation of Approximate Sweetness Equivalents methodology**

**Supplemental Table 1** shows the foods/beverages that were flagged as containing low-calorie sweeteners and the matched pairs used to estimate their ASE values. The table is sorted by the number of times each food/beverage was reported so that the most important/impactful items are near the top. For tabletop sweeteners the values were calculated in-line with published estimates. **Supplemental Table 2** identifies the foods/beverages that were reformulated during the study period and the year in which the reformulation occurred. It is also sorted by number of consumption reports so that the most important/impactful items are near the top.

**Supplemental Table 1.** Foods containing low-calorie sweeteners and their respective matched pairs

| **Items containing low-calorie sweeteners** | | |  | **Matched items** | |
| --- | --- | --- | --- | --- | --- |
| Food Number | Food Name | # of reports |  | Food Number (match) | Food Name (Match) |
|  |  |  |  |  |  |
| 6963 | ROBINSONS NAS FRUIT SINGLE STRENGTH CONCENTRATES, INC BLACKCURRANT | 1985 |  | 6961 | ROBINSONS SINGLE STRENGTH FRUIT DRINK CONCENTRATES ONLY, NOT NAS, INC BLACKCURRANT |
| 2351 | FRUIT DRINK CONC LOW CALORIE NOT BLACKCURRANT | 1687 |  | 6961 | ROBINSONS ORIGINAL FRUIT CONCENTRATES, NOT NAS |
| 2209 | TABLE TOP SWEETENERS IN TABLETS OR MINI CUBES | 1118 |  |  | calculated |
| 10231 | FRUIT DRINK DOUBLE CONCENTRATED, NO ADDED SUGAR, NOT BLACKCURRANT | 828 |  | 6961 | ROBINSONS ORIGINAL FRUIT CONCENTRATES, NOT NAS |
| 8464 | BLACKCURRANT SINGLE STRENGTH FRUIT DRINK CONCENTRATE, NAS, NOT ROBINSONS | 648 |  | 7915 | SINGLE STRENGTH FRUIT CONCENTRATE, NOT NAS, BLACKCURRANT, NOT ROBINSONS, SUGAR~10.9G/100G |
| 7897 | COLA LOW CALORIE NOT CANNED NOT CAFFEINE FREE | 561 |  | 7895 | COLA NOT CANNED NOT LOW CALORIE NOT CAFFEINE FREE |
| 10455 | FRUIT DRINK DOUBLE CONCENTRATED, NO ADDED SUGAR, CONTAINS BLACKCURRANT | 413 |  | 7915 | SINGLE STRENGTH FRUIT CONCENTRATE, NOT NAS, BLACKCURRANT, NOT ROBINSONS, SUGAR~10.9G/100G |
| 7896 | COLA CHERRY COLA LOW CALORIE OR ZERO CANNED NOT CAFFEINE FREE | 361 |  | 7894 | COLA CHERRY COLA CANNED NOT LOW CALORIE |
| 2701 | YOGURT, VIRTUALLY FAT FREE, FRUIT WITH ARTIFICIAL SWEETNER | 255 |  | 11147 | YOGURT VIRTUALLY FAT FREE, FRUIT, STRAINED WITHOUT CREAM |
| 9612 | ARTIFICIAL SWEETENER GRANULATED PER 100G (RECIPES) | 197 |  |  | calculated |
| 7899 | LEMONADE LOW CALORIE NOT CANNED | 183 |  | 2322 | LEMONADE NOT LOW CALORIE NOT CANNED |
| 6959 | ROBINSONS FRUIT SHOOT, NAS, RTD | 180 |  | 6957 | ROBINSONS FRUIT SHOOT, NOT NAS, RTD |
| 5500 | RIBENA REALLY LIGHT NO ADDED SUGAR CONC, NOT RIBENA LIGHT BERRY BURST | 154 |  | 5501 | RIBENA B'CURRANT JUICE DRINK RTD NOT LIGHT |
| 9364 | TABLE TOP SWEETENERS PER 100G | 101 |  |  | calculated |
| 10037 | SINGLE STRENGTH HIGH JUICE DRINK CONCENTRATE, NAS, NOT BLACKCURRANT | 90 |  | 7911 | SINGLE STRENGTH HIGH JUICE FRUIT CONCENTRATE, NOT NAS, NOT BLACKCURRANT |
| 7970 | CHEWING GUM SUGAR FREE | 83 |  | 2253 | CHEWING GUM NOT SUGAR FREE |
| 5110 | SINGLE STRENGTH FRUIT AND BARLEY CONCENTRATE, NOT BLACKCURRANT, NOT ROBINSONS, NAS | 68 |  | 8491 | BARLEY WATER ANY CONC NOT LOW CALORIE |
| 7903 | CARBONATED BEVERAGES NO JUICE LOW CAL NOT CANNED | 64 |  | 7901 | CARBONATED BEVS NO JUICE NOT LOW CAL NOT CANNED |
| 8445 | FRUIT JUICE DRINK <50% JUICE LOW CAL NOT CANNED | 61 |  | 8328 | CARBONATED DRINK <50% JUICE NOT LOW CAL |
| 8380 | TONIC WATER SLIMLINE NOT CANNED | 59 |  | 8378 | TONIC WATER NOT SLIMLINE NOT CANNED |
| 10493 | ACTIMEL PROBIOTIC YOGURT DRINK 0.1% FAT | 56 |  | 10466 | ACTIMEL PROBIOTIC DRINKING YOGURT |
| 5253 | MINERAL WATER STILL OR CARB WITH ARTI SWEETENER | 55 |  | 5343 | MINERAL WATER BASED DRINKS STILL OR CARB; WITH ADDED SUGAR |
| 9272 | YOGURT, VIRTUALLY FAT FREE, ANY OTHER FLAVOUR, WITH ARTIFICAL SWEETENER | 55 |  | 11147 | YOGURT VIRTUALLY FAT FREE, FRUIT, STRAINED WITHOUT CREAM |
| 9996 | FRUIT DRINK CONC BLACKCURRANT NOT LOW CALORIE, ASDA OR SAINSBURY OWN BRANDS ONLY | 44 |  | 5498 | RIBENA ORIGINAL BLACKCURRANT DRINK CONC |
| 10036 | SINGLE STRENGTH HIGH JUICE DRINK CONCENTRATE, NAS, BLACKCURRANT | 43 |  | 7915 | SINGLE STRENGTH FRUIT CONCENTRATE, NOT NAS, BLACKCURRANT, NOT ROBINSONS, SUGAR~10.9G/100G |
| 8474 | BLACKCURRANT JUICE DRINK RTD LOW CALORIE | 40 |  | 8455 | BLACKCURRANT JUICE DRINK RTD NOT LOW CALORIE |
| 9994 | 7-UP LIGHT LOW CALORIE BOTTLED | 34 |  | 9991 | 7 UP CANNED NOT LOW CAL |
| 3895 | CRANBERRY BASED JUICE DRINK; RTD, REDUCED SUGAR/NAS INC CRANBERRY AND ANOTHER JUICE | 31 |  | 3846 | CRANBERRY FRUIT JUICE DRINK EG OCEAN SPRAY |
| 8326 | IRN BRU LOW CALORIE CANNED | 31 |  | 8324 | IRN BRU NOT LOW CALORIE CANNED |
| 5113 | RTD FRUIT FLAVOUR LOW CAL DRINK | 30 |  | 5503 | RIBENA JUICE DRINK RTD, APPLE, STRAWBERRY, NOT CANNED |
| 8327 | IRN BRU LOW CALORIE NOT CANNED | 29 |  | 8325 | IRN BRU NOT LOW CALORIE NOT CANNED |
| 5505 | RIBENA NO ADDED SUGAR BLACKCURRANT RTD LOW CALORIE | 28 |  | 5501 | RIBENA B'CURRANT JUICE DRINK RTD NOT LIGHT |
| 10256 | FLAVOURED MILK DRINKS, NAS, MADE WITH SEMI-SKIMMED MILK | 27 |  | 8212 | MILK DRINK PASTEURISED/STERILISED CHOCOLATE FLAVOUR |
| 20047 | SWEETENERS, INULIN & OLIGOFRUCTOSE | 27 |  |  | calculated |
| 8472 | CITRUS/PINEAPPLE JUICE DRINK RTD LOW CALORIE | 26 |  | 8453 | CITRUS/PINEAPPLE DRINK RTD NOT LOW CALORIE |
| 11352 | STEVIA BASED SWEETENER (CANDEREL) | 26 |  |  | calculated |
| 9101 | TOMATO KETCHUP-REDUCED SALT/SUGAR | 24 |  | 2448 | TOMATO KETCHUP |
| 11320 | SWEETENER (SORBITOL) | 24 |  |  | calculated |
| 11310 | MULLER LIGHT FAT FREE YOGURT WITH ADDED CALCIUM | 22 |  | 704 | YOGURT LOW FAT FRUIT |
| 7902 | CARBONATED BEVERAGE NO JUICE CANNED LOW CALORIE | 19 |  | 7900 | CARBONATED BEVERAGES NO JUICE NOT LOW CAL CANNED |
| 8029 | MIXED / SUMMER FRUIT JUICE DRINK RTD LOW CALORIE | 19 |  | 2358 | MIXED FRUIT JUICE DRINKS, RTD, NOT LOW CALORIE, 25-49% FRUIT |
| 8322 | COLA CHERRY COLA LOW CALORIE CAFFEINE FREE CANNED | 18 |  | 7894 | COLA CHERRY COLA CANNED NOT LOW CALORIE |
| 8323 | COLA LOW CALORIE CAFFEINE FREE NOT CANNED | 17 |  | 7895 | COLA NOT CANNED NOT LOW CALORIE NOT CAFFEINE FREE |
| 10499 | ORANGE JUICE DRINK NAS WITH VIT C AND CALCIUM | 17 |  | 10366 | ORANGE JUICE PASTEURISED FORTIFIED WITH CALCIUM |
| 10294 | LUCOZADE SPORT HYDROACTIVE | 16 |  | 3484 | LUCOZADE SPORT, BOTTLED |
| 8360 | CARBONATED DRINK <50% JUICE LOW CAL CANNED | 15 |  | 8328 | CARBONATED DRINK <50% JUICE NOT LOW CAL |
| 11159 | RIBENA PLUS ACE CONCENTRATE BLACKCURRANT CONC | 15 |  | 5498 | RIBENA ORIGINAL BLACKCURRANT DRINK CONC |
| 11208 | YOGURT VIRTUALLY FAT FREE FRUIT WITH SWEETENERS FORTIFIED | 14 |  | 11147 | YOGURT VIRTUALLY FAT FREE, FRUIT, STRAINED WITHOUT CREAM |
| 9992 | 7 UP LIGHT LOW CAL CANNED | 12 |  | 9991 | 7 UP CANNED NOT LOW CAL |
| 10310 | TESCO FRUIT JUICE DRINK RTD FORTIFIED | 12 |  | 2358 | MIXED FRUIT JUICE DRINKS, RTD, NOT LOW CALORIE, 25-49% FRUIT |
| 3896 | APPLE JUICE DRINK RTD LOW CALORIE/NAS | 11 |  | 8691 | APPLE JUICE DRINK RTD NOT LOW CALORIE |
| 4176 | RED BULL SUGAR FREE | 11 |  | 5545 | RED BULL STIMULATION, CANNED ONLY |
| 8299 | LIQUID TABLE TOP SWEETENERS | 11 |  |  | calculated, estimate |
| 7898 | CARBONATED LEMONADE CANNED LOW CALORIE | 10 |  | 2321 | LEMONADE CANNED NOT LOW CALORIE NOT 7 UP OR SPRITE |
| 9998 | CONC BARLEY WATER LOW CAL BLACKCURRANT, NOT ROBINSONS FRUIT AND BARLEY | 9 |  | 8491 | BARLEY WATER ANY CONC NOT LOW CALORIE |
| 10680 | ALDI FRUIT SHOTS JUICE DRINKS RTD FORTIFIED | 9 |  | 2358 | MIXED FRUIT JUICE DRINKS, RTD, NOT LOW CALORIE, 25-49% FRUIT |
| 11353 | POLYDEXTROSE | 9 |  |  | calculated |
| 7985 | FROMAGE FRAIS VIRT FAT FREE, FRUIT WITH ARTIF SWEETNER | 8 |  | 7739 | FROMAGE FRAIS VIRTUALLY FAT FREE, FRUIT, NO ARTFICIAL SWEETNER |
| 8379 | TONIC WATER SLIMLINE CANNED | 8 |  | 8332 | TONIC WATER NOT SLIMLINE CANNED |
| 2208 | GRANULATED TABLE TOP SWEETENERS | 7 |  |  | calculated |
| 3068 | RICE PUDDING CANNED LOW CAL ARTIFICIAL SWEETENER | 7 |  | 559 | RICE PUDDING CANNED |
| 11135 | MONSTER ABSOLUTELY ZERO ENERGY DRINK | 7 |  | 10527 | MONSTER ORIGINAL ENERGY DRINK |
| 8610 | POTATO CRISPS VERY LOW FAT WITH ARTIFICIAL SWEETENER | 6 |  | 10002 | POTATO CRISPS, PREMIUM, FRIED IN VEGETABLE OIL, EG PRINGLES GOURMET |
| 10388 | ASDA GREAT STUFF FLAVOURED MINERAL WATERS WITH VIT C | 6 |  | 5343 | MINERAL WATER BASED DRINKS STILL OR CARB; WITH ADDED SUGAR |
| 3554 | MILKSHAKE WITH SKIMMED MILK + ARTIFICIAL SWEETENERS | 4 |  | 8215 | MILKSHAKE PURCHASED MADE WITH SEMI-SKIMMED MILK |
| 7919 | C-VIT CONC BLACKCURRANT | 4 |  | 10831 | VITAMIN WATER ESSENTIAL FORTIFIED |
| 10167 | LAZYTOWN FLAVOURED SPRING WATERS NAS FORTIFIED | 4 |  | 10831 | VITAMIN WATER ESSENTIAL FORTIFIED |
| 10763 | MINERAL WATER NAS FRUIT FLAVOUR WITH SWEETENER | 4 |  | 5343 | MINERAL WATER BASED DRINKS STILL OR CARB; WITH ADDED SUGAR |
| 10895 | RIBENA PLUS ACE BLACKCURRANT NAS JUICE DRINK FORTIFIED | 4 |  | 5501 | RIBENA B'CURRANT JUICE DRINK RTD NOT LIGHT |
| 8362 | CARBONATED APPLE JUICE DRINK LOW CALORIE CANNED | 3 |  | 2641 | APPLE JUICE DRINK CARBONATED CANNED NOT LOW CALORIE |
| 10496 | V WATER SHIELD LEMON AND LIME FORTIFIED | 3 |  | 10831 | VITAMIN WATER ESSENTIAL FORTIFIED |
| 11158 | RIBENA PLUS MIXED BERRIES NAS DRINK WITH CALCIUM & VITAMIN C | 3 |  | 5503 | RIBENA JUICE DRINK RTD, APPLE, STRAWBERRY, NOT CANNED |
| 11243 | VITAMIN HIT JUICE DRINK WITH SWEETENER FORTIFIED | 3 |  | 2358 | MIXED FRUIT JUICE DRINKS, RTD, NOT LOW CALORIE, 25-49% FRUIT |
| 2684 | SUGAR FREE ANGEL DELIGHT DRY WEIGHT | 2 |  | 3179 | ANGEL DELIGHT SEMI SKIMMED MILK |
| 3686 | MEATBALLS IN TOMATO SAUCE WITH ARTIFICIAL SWEETENER | 2 |  | 6168 | PORK MEATBALLS IN TOMATO SAUCE |
| 7006 | ALCOHOL SOFT DRINKS, SPIRIT BASED, DIET/NAS E.G BARCARDI BREEZER DIET | 2 |  | 5396 | ALCOHOLIC SOFT DRINKS SPIRIT BASED |
| 8457 | APPLE JUICE DRINK CARBONATED LOW CALORIE | 2 |  | 2641 | APPLE JUICE DRINK CARBONATED CANNED NOT LOW CALORIE |
| 9110 | BOOTS SUCROSE FREE CHOCCO BAR | 2 |  | 2254 | MILK CHOCOLATE BAR |
| 11277 | WATER, SPRING, WITH SWEETENER, VITAMINS AND MINERALS, GLACEAU BRAND | 2 |  | 10831 | VITAMIN WATER ESSENTIAL FORTIFIED |
| 11410 | BOOTS SHAPERS WATERVITS MULTIVITAMIN STILL MINERAL WATER WITH SWEETENER, FORTIFIED | 2 |  | 10831 | VITAMIN WATER ESSENTIAL FORTIFIED |
| 3257 | SUNNY D, NO ADDED SUGAR JUICE DRINK, FORMERLY SUNNY DELIGHT | 1 |  | 6827 | SUNNY D FRUIT JUICE DRINK, FORMERLY SUNNY DELIGHT |
| 7226 | CHOCOLATE ENERGY AND PROTEIN BARS, FORTIFIED, WITH SWEETENERS EG ATKINS ADVANTAGE | 1 |  | 2254 | MILK CHOCOLATE BAR |
| 10169 | DIET COKE PLUS VITAMINS | 1 |  | 7895 | COLA NOT CANNED NOT LOW CALORIE NOT CAFFEINE FREE |
| 10495 | V WATER KICK GINGER AND MANGO FORTIFIED | 1 |  | 10831 | VITAMIN WATER ESSENTIAL FORTIFIED |
| 10840 | VITABIOTICS WELLWOMAN DRINK | 1 |  | 10831 | VITAMIN WATER ESSENTIAL FORTIFIED |
| 11288 | GET MORE MANGO AND PASSION FRUIT DRINK WITH ADDED VITAMIN D AND CALCIUM | 1 |  | 9157 | RUBICON MANGO JUICE DRINK RTD |

Acronyms: NAS = no added sugar; RTD = ready to drink

**Supplemental Table 2.** Foods and beverages undergoing reformulation with the addition of LCS and the year in which reformulation occurred

| Food Number | Food Name | Reformulation year | # of reports |
| --- | --- | --- | --- |
|  |  |  |  |
| 2322 | LEMONADE NOT LOW CALORIE NOT CANNED | 2014-15 | 187 |
| 10035 | ROBINSONS FRUIT AND BARLEY CONCENTRATE, ANY FLAVOUR | 2009-10 | 177 |
| 7901 | CARBONATED BEVS NO JUICE NOT LOW CAL NOT CANNED | 2018-19 | 165 |
| 2349 | SINGLE STRENGTH FRUIT DRINK CONC, NOT B/C, NOT NAS, NOT ROBINSONS HIGHER SUGAR ~33G/100G SUGAR | 2015-16 | 146 |
| 5498 | RIBENA ORIGINAL BLACKCURRANT DRINK CONC | 2017-18 | 131 |
| 2358 | MIXED FRUIT JUICE DRINKS, RTD, NOT LOW CALORIE, 25-49% FRUIT | 2017-18 | 125 |
| 8453 | CITRUS/PINEAPPLE DRINK RTD NOT LOW CALORIE | 2017-18 | 123 |
| 3893 | LUCOZADE ENERGY INC ORIGINAL, ORANGE & LEMON VARIETIES. NOT CANNED | 2016-17 | 112 |
| 8325 | IRN BRU NOT LOW CALORIE NOT CANNED | 2018-19 | 109 |
| 7911 | SINGLE STRENGTH HIGH JUICE FRUIT CONCENTRATE, NOT NAS, NOT BLACKCURRANT | 2015-16 | 99 |
| 7912 | MIXED FRUIT JUICE DRINK, RTD, NOT LOW CAL, NOT BLACKCURRANT, 50-70% FRUIT | 2015-16 | 91 |
| 2340 | FRUIT JUICE DRINK CARB LESS THAN 50% JUICE NOT CANNED | 2018-19 | 82 |
| 8144 | FRUIT JUICE DRINK WITH 5% FRUIT JUICE RTD | 2017-18 | 76 |
| 8378 | TONIC WATER NOT SLIMLINE NOT CANNED | 2014-15 | 75 |
| 7890 | INSTANT LOW CALORIE CHOCOLATE DRINK DRY WT | 2016-17 | 71 |
| 3846 | CRANBERRY FRUIT JUICE DRINK WITH ADDED VITAMIN C EG OCEAN SPRAY | 2017-18 | 67 |
| 8324 | IRN BRU NOT LOW CALORIE CANNED | 2017-18 | 67 |
| 8888 | LUCOZADE SPORT ISOTONIC DRINK NOT CARBONATED | 2015-16 | 63 |
| 8455 | BLACKCURRANT JUICE DRINK RTD NOT LOW CALORIE | 2017-18 | 59 |
| 5501 | RIBENA B'CURRANT JUICE DRINK RTD NOT LIGHT | 2017-18 | 58 |
| 7913 | SINGLE STRENGTH FORTIFIED HIGH JUICE FRUIT CONCENTRATE, NOT NAS, BLACKCURRANT | 2015-16 | 56 |
| 5343 | MINERAL WATER BASED DRINKS STILL OR CARB; WITH ADDED SUGAR | 2014-15 | 55 |
| 8328 | CARBONATED DRINK <50% JUICE NOT LOW CAL | 2017-18 | 54 |
| 8691 | APPLE JUICE DRINK RTD NOT LOW CALORIE | 2016-17 | 51 |
| 7900 | CARBONATED BEVERAGES NO JUICE NOT LOW CAL CANNED | 2014-15 | 49 |
| 8210 | PLJ LEMON JUICE CONCENTRATE WITH VITAMIN C | 2015-16 | 43 |
| 10166 | BOOST ENERGY DRINK WITH B VITS | 2016-17 | 39 |
| 5503 | RIBENA JUICE DRINK RTD, APPLE, STRAWBERRY, NOT LIGHT, NOT CANNED | 2015-16 | 31 |
| 9157 | RUBICON MANGO JUICE DRINK RTD | 2018-19 | 31 |
| 2321 | LEMONADE CANNED NOT LOW CALORIE NOT 7 UP OR SPRITE | 2014-15 | 29 |
| 9993 | 7-UP NOT CANNED NOT LOW CALORIE | 2018-19 | 25 |
| 11256 | CRANBERRY FRUIT JUICE DRINK NO ADDED VITAMIN C | 2017-18 | 25 |
| 2670 | OVALTINE LIGHT INSTANT LOW FAT DRY WEIGHTS | 2009-10 | 18 |
| 10261 | DALE FARM SUPA SUKIE ORANGE FRUIT DRINK FORTIFIED | 2015-16 | 18 |
| 8399 | TAUT ISOTONIC ENERGY DRINK FORTIFIED | 2016-17 | 17 |
| 9991 | 7 UP CANNED NOT LOW CAL | 2017-18 | 15 |
| 10158 | ICED TEA FRUIT FLAVOURED | 2016-17 | 14 |
| 711 | YOGURT DRINK | 2016-17 | 11 |
| 2320 | APPLE JUICE DRINK CARBONATED NOT LOW CAL NOT CANNE | 2009-10 | 11 |
| 10460 | ROCKSTAR ENERGY DRINK | 2017-18 | 11 |
| 9799 | ENSURE LIQUID | 2017-18 | 10 |
| 8332 | TONIC WATER NOT SLIMLINE CANNED | 2014-15 | 7 |
| 3548 | FRUIT JUICE DRINK WITH VIT C EG. SUN EXOTIC | 2017-18 | 4 |
| 11043 | FRUIT/VEGETABLE JUICE DRINKS FORTIFIED ACE NOT LOW CAL EG PURITY DRINKS JUICE BURST | 2017-18 | 4 |

Acronyms: NAS = no added sugar; RTD = ready to drink

| **Supplemental Table 3.** Example of implementation of ASE approximation algorithm using a single day of data for a hypothetical individual for 2018-19 | | | | | | |
| --- | --- | --- | --- | --- | --- | --- |
| **Time** | **FoodNumber** | **FoodName** | **Grammes** | **Kilocalories** | **Total Sugar, grammes** | **Approximate Sugar Equivalents, grammes** |
| 8:30 AM | 126 | BREAD WHITE TOASTED | 65.9 | 177.9 | 2.6 | 2.6 |
| 8:30 AM | 10040 | FAT SPREAD (62-72% FAT) NOT POLYUNSATURATED | 18.0 | 114.1 | 0.1 | 0.1 |
| 8:30 AM | 2315 | TEA WEAK INFUSION | 230.0 | 0 | 0 | 0.0 |
| 8:30 AM | 608 | MILK SEMI-SKIMMED PASTEURISED SUMMER | 106.8 | 47.0 | 4.2 | 4.2 |
| 12:30 PM | 7900  (example 1) | CARBONATED BEVERAGES NO JUICE NOT LOW CAL NOT CANN | 284.0 | 58.0 | 14.5 | **23.9** |
| 12:30 PM | 1333 | CHEESEBURGER TAKEAWAY NOT QTER POUNDER | 115.0 | 292.1 | 5.6 | 5.6 |
| 12:30 PM | 1876 | CHIP FROZEN STRAIGHT CUT COMMERCIAL BLENDED OIL | 82.5 | 193.0 | 0.7 | 0.7 |
| 12:30 PM | 1789 | ONIONS FRIED BLENDED VEGETABLE OIL | 30.0 | 70.2 | 2.9 | 2.9 |
| 12:30 PM | 2448 | TOMATO KETCHUP | 16.0 | 17.3 | 3.7 | 3.7 |
| 3:00 PM | 2349 | FRUIT DRINK CONC NOT B/C NOT LOW CAL, NOT JS OR ASDA | 2349.0 | 57.0 | 49.0 | 49.0 |
| 3:00 PM | 5101 | WATER FOR CONCENTRATED SOFT DRINKS NOT DIET CODE 5101 | 5101.0 | 240.0 | 0.0 | 0.0 |
| 3:00 PM | 1713 | CARROTS, YOUNG, FRESH, BOILED | 320.0 | 70.4 | 13.4 | 13.4 |
| 7:00 PM | 1090 | CHICKEN ROAST LIGHT MEAT ONLY | 30.0 | 45.9 | 0.0 | 0.0 |
| 7:00 PM | 8744 | CIABATTA WHITE BREAD MADE WITH OLIVE OIL | 100.0 | 272.0 | 0.4 | 0.4 |
| 7:00 PM | 7896  (example 2) | COLA CHERRY COLA LOW CALORIE OR ZERO CANNED NOT CAFFEINE FREE | 250.0 | 2.5 | 0.0 | **28.3** |
| 7:00 PM | 1762 | LETTUCE UNSPECIFIED RAW | 13.0 | 1.8 | 0.2 | 0.2 |
| 7:00 PM | 2432 | MAYONNAISE (RETAIL) | 8.0 | 55.2 | 0.1 | 0.1 |
| 7:00 PM | 3991 | POTATO WEDGES BAKED | 165.0 | 297.0 | 1.9 | 1.9 |
| 7:00 PM | 908 | RASHERS CUT UNSPEC NOT SMOKED GRILLED LEAN AND FAT | 25.0 | 75.3 | 0.0 | 0.0 |
| 7:00 PM | 1931 | CHICKEN ROAST LIGHT MEAT ONLY | 22.0 | 3.7 | 0.7 | 0.7 |
| 10:00 PM | 2402 | 70% PROOF SPIRITS WHISKY GIN BRANDY RUM VODKA BACARDI MALIBU | 23.0 | 53.8 | 0.0 | 0.0 |
| 10:00 PM | 7897  (example 3) | COLA LOW CALORIE NOT CANNED NOT CAFFEINE FREE | 110.0 | 1.1 | 0.0 | **12.4** |
| 10:00 PM | 8198 | CRUNCH BISCUIT WITH CREAM FILLING | 13.0 | 65.5 | 5.3 | 5.3 |
| **Total** | **-** | **-** | **9477** | **2211** | **105.3** | **155.4** |

**Worked example.** This hypothetical individual reported consuming 23 items, 9477 grammes and 2211 kilocalories. Their total sugar intake was estimated to be 105.3 g/d. Because they consumed 3 items that contained low-calorie sweeteners, they also had their ASE values estimated. Example 1 was a food that underwent reformulation during the study period, and which we confirmed LCS were used in addition to caloric sweeteners in later years. The original amount of sugar per 100g for this item before reformulation was 9.4g and the estimated value in 2017-18 was 5.4, therefore the ASE value was estimated to the be (2.84 * 9.4 = 26.7). The other two examples are diet/low-calorie beverages that did not undergo reformulation. These two items both had respective ASE values of 11.3g per 100g and therefore have estimated values of 28.3 and 12.4 grammes ASE, respectively. Had the individual not consumed these three items their ASE value would have been the same as their total sugars value.

**Part II. Sensory Trial Methods**

*Samples*

The list of sweet tasting product categories were chosen based on those significantly contributing to overall LCS consumption (in terms of frequency) from NDNS 2008/09-2018/19, capturing products that represent about 74% of ASE from LCS in the UK diet. The specific matched sweet tasting products (full sugar vs. LCS equivalent) were identified across different large national brands. The list of products is shown in **Supplemental Table 1**. The products were purchased by Sensory Dimensions Ltd. (Reading, UK) and any products that needed prep were prepared according to manufacturer’s instructions. All beverages were served cold and 100ml sample was served to each panelist.

*Procedure*

The samples were analyzed for sweetness intensity by 11 members of the Sensory Dimensions Panel, trained and experienced in flavor evaluation. The strength of each attribute was rated on the 15-point Spectrum Scale, where 0 = none and 15 = very strong.​This scale incorporates the ability to use tenths of a point and therefore has the potential of 150 scale differentiations (Hootman 1992). The panelists evaluated each sample in a randomized order and recorded individual ratings for each attribute in RedJade® software (RedJade Sensory Solutions, LLC, Martinez, CA). All samples were expectorated. Panelists cleared with water in between each sample as well as unsalted crackers, if needed. The next sample was brought out when all panelists had had enough time to clear.

*Data Analysis*

Univariate Analysis of Variance (ANOVA) was used to analyze differences in in-mouth sweet taste (highest point) between matched samples. The model included sample (fixed effect) and panelist (random effect). Significant differences among the samples are reported at both the 95% and the 90% confidence levels. ​Sample means were then compared using Fisher’s Least Significant Differences (95% CL and 90% CL) to identify which samples differ from each other. XLStat Statistical Software for Excel (version 2021.4.1.1198) was used to run the PCA analysis and the histograms; SenPAQ was used to run the ANOVA and post hoc test (Senpaq version 5.01, Qi Statistics, UK). The significance level was set at α < 0.05 and < 0.10 for 95% and 90% CL respectively for the ANOVA calculations.

**Sensory Trial Results**

Results of average in-mouth sweet taste (highest point) assessment of matched (full sugar vs. LCS equivalent) sweet tasting products are shown in **Supplemental Table 1**. No significant differences were observed in average in-mouth sweet taste between matched (full sugar vs. LCS equivalent) products. In our previous research (1) no significant differences were observed for sweeteners (aspartame, sucralose, saccharin, stevia vs sugar). This suggests, that differences in sweetness are generally comparable across different products and categories.

**Supplemental Table 4.** Differences in average in-mouth sweet taste (highest point) between matched (full sugar vs. LCS equivalent) sweet tasting products

| **Alias name** | **Brand name** | **Full sugar** | **LCS equivalent** | **p-value of difference** |
| --- | --- | --- | --- | --- |
| **Beverages** | | | | |
| **Carbonated soft drinks** |  |  |  |  |
| Cola | Coke Regular vs. Diet Coke | 6.2 | 6.3 | 0.836 |
| Lemonade | 7 up Regular vs. 7 up Free | 6.7 | 7.1 | 0.254 |
| **Fruit drinks** |  |  |  |  |
| Juice drink | Ribena Regular vs Ribena Light | 6.1 | 6.4 | 0.257 |
| **Energy drinks** |  |  |  |  |
| Energy drink | Red Bull vs. Red Bull Sugar Free | 7.0 | 6.9 | 0.865 |
| **Foods** | | | | |
| Yogurt | Müller Strawberry Corner vs. Müller Light Strawberry | 6.2 | 6.5 | 0.339 |

**References**

1. Kamil A, Wilson, A.R., Rehm, C.D. Estimated Sweetness in US Diet Among Children and Adults Declined From 2001 to 2018: A Serial Cross-Sectional Surveillance Study Using NHANES 2001-2018. Front Nutr. 2021;8.
